# Comprehensive Surveillance of Severe Fever with Thrombocytopenia Syndrome Virus in Patients with Acute Febrile Illness, Wild Rodents, and Trombiculid Larval Mites, Thailand

**DOI:** 10.3201/eid3014.240163

**Published:** 2024-11

**Authors:** Piyada Linsuwanon, Yong Poovorawan, Keun Hwa Lee, Nutthanun Auysawasdi, Sirima Wongwairot, Chawin Limsuwan, Viboonsak Vuthitanachot, Surachai Leepitakrat, Sompong Vongpunsawasdi, Pornjarim Nilyanimit, Yossapong Paladsing, Erica Lindroth

**Affiliations:** Walter Reed Army Institute of Research–Armed Forces Research Institute of Medical Sciences, Bangkok, Thailand (P. Linsuwanon, N. Auysawasdi, S. Wongwairot, C. Limsuwan, S. Leepitakrat, Y. Paladsing, E. Lindroth); Chulalongkorn University Faculty of Medicine, Bangkok (Y. Poovorawan, S. Vongpunsawasdi, P. Nilyanimit); Hanyang University, Seoul, South Korea (K.H. Lee); Chum Phae Hospital, Khon Kaen, Thailand (V. Vuthitanachot)

**Keywords:** severe fever with thrombocytopenia syndrome virus, Bandavirus, viruses, Bandavirus dabieense, Phenuiviridae, severe fever with thrombocytopenia syndrome, SFTS, SFTSV, acute febrile illness patients, rodent, chigger, Thailand, vectorborne infections

## Abstract

Infection with severe fever with thrombocytopenia syndrome (*Bandavirus dabieense*) virus poses a substantial public health threat because of its high mortality rates and severe complications. The virus is prevalent in Asia, although data from Thailand are scarce. Our study confirmed the virus in 1.6% of acute febrile illness patients and specific antibodies in 3% of archived samples since 2015 in Thailand. Nationwide zoonotic surveillance identified the virus in 8 rodent species and 4 chigger genera. Our findings underscore the importance of raising awareness among healthcare providers and the general public about the symptoms, risks, and prevention strategies associated with severe fever with thrombocytopenia syndrome virus infection. Ongoing surveillance of the virus in human and animal populations is essential for monitoring its prevalence, distribution, and potential for emergence.

Severe fever with thrombocytopenia syndrome virus (SFTSV) is a virulent virus with a triple-segmented, negative-sense, single-stranded RNA genome. Taxonomically *Bandavirus dabieense*, the virus belongs to the genus *Bandavirus*, family *Phenuiviridae*. SFTSV poses a substantial public health challenge because of the lack of a vaccine or effective therapies and high mortality rates in previously healthy persons (*1*–*3*). First discovered in China in 2009 (*4*), SFTSV has since been reported in China, South Korea, and Japan and more recently in Vietnam, Myanmar, Pakistan, Taiwan, and Thailand (*5*–*12*). The virus is classified into genotypes A–F, each having distinct geographic variations in virulence and pathogenicity (*13*–*15*).

SFTSV is predominantly transmitted through tick bites, specifically by the Asian longhorned tick *Haemaphysalis longicornis*, known for its wide host range, vector competency for various pathogens, and extensive geographic distribution (*16*). Additional competent vectors include *Haemaphysalis* *flava* (*17*), *Ixodes sinensis* (*18*), and >8 other implicated tick species (*19*,*20*). Tick-bite prevention is considered the primary means of preventing SFTSV infection. Evidence also implicates mites in SFTSV transmission, particularly those in the family *Trombiculidae*, including *Leptotrombidium scutellare* and *Leptotrombidium deliense*, and family *Laelapidae*, including *Laelaps echidninus* (*21*,*22*). 

Human-to-human transmission of SFTSV occurs through direct contact with infected blood or bodily fluids (*23*). Animal-to-human transmission is occasionally reported though contact with ill animals (*12*,*24*). The role of wild and domesticated animals has garnered considerable interest because of their potential involvement as pathogen reservoirs. In addition, the presence of viral RNA or specific antibodies has been confirmed in 10 domestic (*20*,*25*–*27*) and >10 wild animal species (*28*–*30*). This broad host and vector involvement underlines the complex epidemiology of SFTSV, posing major challenges in developing targeted public health strategies and mitigating the effect of this virus. Among vertebrate reservoirs of concern, rodents receive considerable attention because of their close proximity to humans.

Although SFTSV in Thailand was documented in 2020 (*12*), evidence suggests that the presence of SFTSV dates back to 2019 (*31*). Subsequent analysis of patients clinically suspected of having viral infection confirmed the presence of SFTSV genome segments belonging to genotype B (*32*). One study in Thailand investigated dogs as amplifying hosts, and the nucleotide sequence of SFTSV found in 1 dog appears closely related to genotype B or J3 (*33*). Although evidence from human and animal studies indicates the presence of SFTSV in multiple provinces of Thailand, primarily in Bangkok and its neighboring regions (Figure 1), the understanding of its distribution and potential hotspots remains incomplete. Comprehensive surveillance, particularly from regions with extensive farming or agricultural activities, where these habitats could serve as zoogeographic transmission points and disease hotspots, is imperative to guide effective prevention and control strategies.

**Figure 1:**
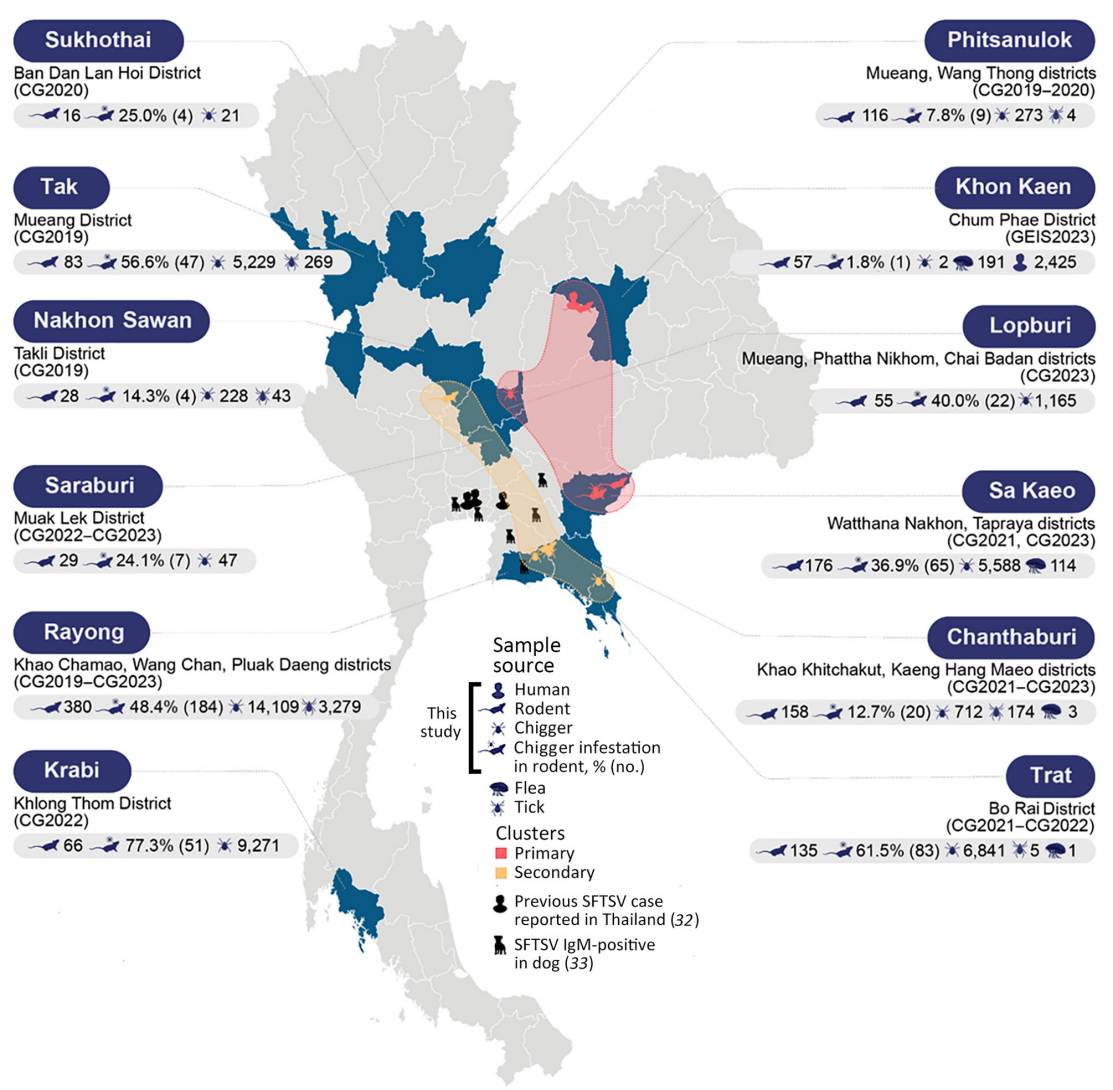
Geospatial clustering of SFTSV, Thailand, 2015–2021. Blue areas on the map represent the surveillance locations in this study. Icons indicate the types of host species and chiggers that tested positive for SFTSV. Data were consolidated to include previous reports of locations where SFTSV-positive patients and dogs were identified, aligning with the current locations of positive samples. Clusters were determined using the K-means clustering method. Primary clusters, highlighted in red, denote regions with a high overall prevalence of SFTSV across all hosts or are considered high-risk areas. Secondary clusters, highlighted in yellow, indicate areas with potential transmission dynamics, particularly involving animal hosts. SFTSV, severe fever with thrombocytopenia syndrome virus.

## The Study

To better understand the epidemiologic effect of SFTSV in Thailand, our study evaluated the prevalence of SFTSV RNA and specific antibodies in serum samples from 2,425 patients with undifferentiated acute febrile illness (AFI). The patients were admitted to Chum Phae Hospital in Khon Kaen Province, northeastern Thailand, during 2015–2021. We performed RNA detection by using quantitative reverse transcription PCR (qRT-PCR) targeting the partial nonstructural (NS) protein–encoding gene of the small segment (Appendix). We subsequently screened all SFTSV RNA-positive samples for other vectorborne pathogens endemic in Southeast Asia, including dengue, chikungunya, and Zika virus, by using the ZDC Multiplex RT-PCR Assay Kit (Bio-Rad, https://www.bio-rad.com) and bacterial pathogens, including *Rickettsia* and *Orientia* spp., by following a previously described protocol (*34*). We verified all qRT-PCR products for the expected size and subjected them to nucleotide sequencing by using the barcode-tagged sequencing method (Bionics, https://www.bionicsro.co.kr). We conducted phylogenetic analysis by using MEGA version 11 (https://www.megasoftware.net). The resulting nucleotide sequences of SFTSV amplified from the samples have been deposited into the GenBank database (accession nos. PP782658–61).

**Figure 2:**
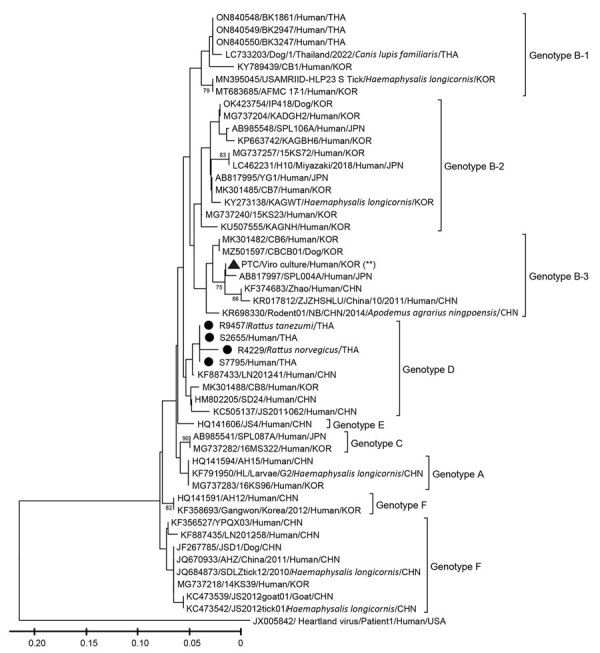
Phylogenetic analysis of the partial sequences of nonstructural protein–encoding gene in the small segment of severe fever with thrombocytopenia syndrome virus identified in Thailand, 2015–2021 and other countries. Black circles indicate nucleotide sequences identified in this study; black triangle indicates nucleotide sequence of the positive control originated from SFTSV patients in South Korea. The sequences are described by GenBank accession numbers/strain names/hosts/3-letter country code. Bootstrap values of nodes, based on 1,000 bootstrapping replicates, associated with the definition of genotypes are indicated in the principal nodes. Scale bar indicates phylogenetic distance of 0.05 nucleotide substitutions per site.

We detected SFTSV RNA in 38 of 2,425 AFI patients, resulting in a positivity rate of 1.6%. The median age of SFTSV RNA–positive patients was 47.2 years (15.9–86.7 years) (first–third interquartile range [IQR_1–3_] 29–64 years). A total of 36.8% of the patients had agriculture-related occupations; male:female ratio was 1.4:1.0 (Table 1). The SFTSV RNA–positive cases did not exhibit a discernible seasonal pattern. We observed no co-positivity between SFTSV and other pathogens. SFTSV RNA–positive patients often visited the hospital with fever (38.0°C [range 36.1°C–40.3°C]); 20.5% experienced headaches, and 12.8% reported dizziness. We observed thrombocytopenia and variations in hemoconcentration in 3 cases (Appendix Figure 1). Of note, all patients recovered without severe complications. We successfully obtained only 2 nucleotide sequences from SFTSV RNA–positive patients, both of which demonstrated a close genetic relationship to genotype D. The strains shared 99.1% nucleotide similarity to strain LN2012–41 (GenBank accession no. KF887433) previously identified in a patient in China in 2012 (Figure 2).

**Table 1:** Demographic disparities in acute febrile illness patients with SFTSV RNA–positive and specific antibodies–positive samples, Thailand, 2015–2021*

Variable	Total no. (%)	SFTSV positive	
		No. (%) qRT-PCR positive	No. (%) ELISA positive
Sex			
F	1,113 (45.9)	10 (26.3)	24 (32.9)
M	1,105 (45.6)	23 (60.5)	17 (23.3)
No information	207 (8.5)	5 (13.2)	32 (43.8)
Age range, y			
>15	1,474 (60.8)	0	0
0–30	584 (24.1)	8 (21.1)	20 (27.4)
31–40	34 (1.4)	7 (18.4)	3 (4.1)
41–50	36 (1.5)	1 (2.6)	3 (4.1)
51–60	38 (1.6)	4 (10.5)	3 (4.1)
61–70	20 (0.8)	5 (13.2)	7 (9.6)
>70	32 (1.3)	6 (15.8)	5 (6.8)
No information	207 (8.5)	7 (18.4)	32 (43.8)
Year			
2015	252 (10.4)	2 (5.3)	0
2016	99 (4.1)	1 (2.6)	0
2017	260 (10.7)	0	0
2018	203 (8.4)	2 (5.3)	15 (20.5)
2019	680 (28)	15 (39.5)	24 (32.9)
2020	486 (20)	1 (2.6)	2 (2.7)
2021	238 (9.8)	11 (28.9)	0
No information	207 (8.5)	6 (15.8)	32 (43.8)
Month			
January	150 (6.2)	2 (5.3)	1 (1.4)
February	154 (6.4)	8 (21.1)	0
March	196 (8.1)	6 (15.8)	0
April	131 (5.4)	0	2 (2.7)
May	100 (4.1)	0	6 (8.2)
June	160 (6.6)	11 (28.9)	6 (8.2)
July	313 (12.9)	0	9 (12.3)
August	370 (15.3)	1 (2.6)	6 (8.2)
September	183 (7.5)	1 (2.6)	1 (1.4)
October	186 (7.7)	0	5 (6.8)
November	168 (6.9)	2 (5.3)	4 (5.5)
December	107 (4.4)	1 (2.6)	1 (1.4)
No information	207 (8.5)	6 (15.8)	32 (43.8)
Season			
Hot, Mar–May	427 (17.6)	6 (15.8)	27 (37)
Rainy, Jun–Oct	1212 (50)	13 (34.2)	6 (8.2)
Winter, Nov–Feb	579 (23.9)	13 (34.2)	8 (11)
No information	207 (8.5)	6 (15.8)	32 (43.8)
Total	2,425	38 (1.6)	73 (3)

*qRT-PCR, quantitative reverse transcription PCR; SFTSV, severe fever with thrombocytopenia syndrome virus.

We used an indirect ELISA specific for nucleoprotein (NP) of SFTSV to detect SFTSV IgM and SFTSV IgG in all 2,425 serum samples of AFI patients, according to manufacturer instructions (Bore Da Biotech, http://boreda.com). The average optical density at 450 nm (OD_450_) of the positive controls provided by the kit was 0.233 for SFTSV IgM and 0.172 for SFTSV IgG. We considered samples with OD_450_ values exceeding those cutoffs to be seropositive (Appendix Figure 2). Serologic analysis revealed that 16 patients (0.7%) were seropositive for SFTSV IgM and 54 patients (2.2%) were seropositive for SFTSV IgG; 3 patients (0.1%) had detectable levels of both antibodies (Appendix Figure 2). Those findings resulted in an overall seropositive rate of 3% among the study population (Table 1). The median age of SFTSV IgM–seropositive patients was 63.8 years (IQR_1–3_ 20.6–68.1 years) and for SFTSV IgG–seropositive patients was 14 years (IQR_1–3_ 8.4–51.5 years). We observed no co-positive results between ELISA and qRT-PCR in the tested samples.

We evaluated the practicality of the paper-based lateral flow immunochromatography rapid test (SFTSV RDT; Bore Da Biotech) for potential use in prescreening of the viral NP at the point-of-care. Of the 38 SFTSV RNA–positive serum samples, 33 samples had sufficient quantities and were included in the analysis. The SFTSV RDT accurately detected the viral NP when the samples had a qRT-PCR cycle threshold of <37 or an average of 2.72 × 10^4^ copies/mL serum observed in our study, demonstrating a positive concordance rate of 89.5% between both assays. Control tests with serum from healthy donors and patient serum samples previously confirmed positive for dengue virus 1–4, chikungunya virus, *Rickettsia typhi*, or *Orientia tsutsugamushi* showed no cross-reactivity. The overall agreement between SFTSV RDT and qRT-PCR was substantial, having a κ value of 0.732, which validated its effectiveness (Appendix Table 3).

To investigate the role of rodents and chiggers in SFTSV transmission, we analyzed a total of 2,052 tissue samples from 1,019 wild rodents, representing 15 species across 7 genera and 4 families, by using qRT-PCR targeting the NS gene. We collected the samples during 2019–2023 as part of the rodentborne and ectoparasiteborne disease risk assessment program in Thailand (Appendix). Eleven rodents from 8 species were positive for SFTSV RNA, indicating an overall positivity rate of 1.1%. We collected the RNA-positive samples during 2019–2023 (Appendix Table 1) in Nakhon Sawan, Chanthaburi, Sa Kaeo, and Khon Kaen Provinces, and the positive rodent species included *Mus caroli* (Ryukyu mouse), *Menetes berdmorei* (Berdmore’s squirrel), *Rattus norvegicus* (Norway rat), *Berylmys berdmorei* (small white-toothed rat), *Rattus exulans* (Polynesian rat), *Bandicota indica* (greater bandicoot rat), *Bandicota savilei* (Savile’s bandicoot rat), and *Rattus tanezumi* complex (Asian house rat) (Table 2). Of the 1,019 rodents analyzed, we observed the highest SFTSV RNA prevalence in lungs (0.4% [4 rodents]) and spleen (0.4% [4 rodents]), followed by kidneys (0.3% [3 rodents]). Phylogenetic analysis of the partial sequence of the NS gene obtained from 2 rodents indicated a close relationship to genotype D, sharing 97.3% and 99.1% nucleotide similarity to the strain LN2012–41 from China and being nearly identical to SFTSV sequences obtained from AFI patients (Figure 2). The high genetic similarity observed across different locations and periods suggests a potential widespread distribution of SFTSV in Thailand. However, this observation might also be influenced by the limitation of using only 124-bp nucleotide sequences for phylogenetic analysis. To ensure the validity of our findings and to rule out the possibility of cross-contamination, we performed nucleic acid extraction of samples and qRT-PCR in 2 separate laboratories by using different stocks of samples and reagents for confirmation. In addition, we prepared the positive control for the assay by using virus culture stock from an SFTSV RNA–positive case in South Korea that displayed a genetic distance from our positive samples.

**Table 2:** Positivity rates of SFTSV RNA detected in wild rodents, Thailand, 2015–2021*

Family	Species	No. (%) positive	SFTSV RNA–positive, by tissue			Average RNA level, copies/mL	
			Lung	Liver	Spleen		
*Muridae*	*Rattus tanezumi* rat	1/559 (0.2)	–	1	–	4.07 × 10^4^	
	*R. exulans* rat	1/98 (1)	–	–	1	3.05 × 10^3^	
	*R. novegicus* rat	1/16 (6.3)	1	–	–	5.73 × 10^3^	
	*Mus cervicolor* mouse	0/6	–	–	–	–	
	*M. caroli* mouse	1/7 (14.3)	1	–	–	4.03 × 10^3^	
	*Bandicota indica* rat	2/113 (1.8)	1	1	–	4.89 × 10^3^	
	*B. savileii* rat	2/121 (1.7)	–	2	–	1.01 × 10^4^	
	*Maxomys surifer* rat	0/23	–	–	–	–	
	*Niviventer fulvescens* rat	0/5	–	–	–	–	
	*Berylmys berdmorei* rat	1/23 (4.3)	–	–	1	1.71 × 10^4^	
	*B. bowersi* rat	0/1	–	–	–	–	
	*Chiromyscus chiropus* rat	0/1	–	–	–	–	
*Tupaiidae*	*Tupaia belangeri* shrew	0/19	–	–	–	–	
	*T. glis* shrew	0/9	–	–	–	–	
*Sciuridae*	*Menetes berdmorei* squirrel	2/19 (10.5)	1	–	1	4.06 × 10^3^	
3 families	15 species	11/11,019 (1.1)	4	4	3		
*SFTSV, severe fever with thrombocytopenia syndrome virus; – , negative result.						

From the analysis of 573 individual chiggers retrieved from 155 wild rodents, we detected SFTSV RNA in 8 chiggers (1.4%), which had an average SFTSV RNA level of 2.40 × 10^4^ copies/chigger (range 5.80 × 10^3^–70 × 10^4^ copies/chigger) (Appendix Table 2). We retrieved the SFTSV RNA–positive chiggers from 6 rodents from Sa Kaeo, Chanthaburi, Lopburi, Rayong, and Trat provinces. Phylogenetic analysis of cytochrome oxidase subunit I gene sequences confirmed the correct genus assignment for SFTSV RNA–positive chiggers and revealed close relatedness to *Gahrliepia* (*walchia*) (4 samples), *Blankaartia acuscutellaris* (1 sample), and *Schoengastia kanhaensis* mites (1 sample) (Appendix Figure 3). Two SFTSV RNA–positive chiggers from the genus *Leptotrombidium* had insufficient sample quantities for further analysis. In addition, 4 of 8 SFTSV RNA–positive chiggers showed co-positivity for unidentified *Rickettsia*, but none tested positive for *O. tsutsugamushi*.

## Discussion

Our study confirmed the presence of SFTSV RNA in AFI patients, wild rodents, and chiggers across multiple locations in Thailand and identified a notable seroprevalence of SFTSV-specific antibodies among AFI patients, highlighting a substantial yet underrecognized prevalence of SFTSV. The analysis of samples spanning multiple years, including the detection of SFTSV RNA in AFI patients in 2015, suggests that SFTSV has been circulating in Thailand since 2015, which predates the SFTSV detections reported in other Southeast Asia countries, including Vietnam in 2017 (*35*), Thailand in 2019 (*12*,*31*) and Myanmar during 2018–2019 (*11*). Our findings provide additional evidence of the existence of SFTSV genotype D, indicating that >2 genotypes have been identified recently in Thailand. Positive cases in patients and animals have been identified across several provinces in multiple regions, including Chachoengsao, Samutprakan, and Chonburi Provinces in the central region; Nakhon Sawan Province in the northern region; Chanthaburi, Sa Kaeo, Prachinburi, Rayong, and Chonburi Provinces in the eastern region; and Khon Kaen Province in the northeastern region (Figure 1) (*31*–*33*). This extensive distribution of the virus signifies a widespread and longstanding effect, necessitating ongoing surveillance and enhanced diagnostic measures to fully comprehend the disease ecology and transmission dynamics and to manage public health interventions effectively.

We observed that the prevalence of SFTSV RNA in wild rodents in our study was similar to the rate reported in China, where 0.7% of rodents tested positive. The species in China included *Apodemus agrarius* (striped field mouse), *Crocidura lasiura* (Ussuri white-toothed shrew), *R. norvegicus* (brown rat), and *Mus musculus* (house mouse) (*22*). A subsequent study from the same authors reported a higher prevalence of 32.3% and positive species including *A. agrarius* mice, *Tscherskia triton* (greater long-tailed hamster), and *M. musculus* mice (*14*). The primary difference between our study and previous studies lies in the surveillance sites and periods. In our study, rodents were captured from semirural areas near communities and were primarily collected during the dry season in Thailand within a 2–3-month timeframe at selected sites. During this period, agricultural activities in some locations are reduced, which could potentially limit rodent abundance and their exposure to a broader range of ectoparasites, including ticks. To more accurately determine whether rodents serve as reservoirs of SFTSV and better understand the complex dynamics of SFTSV transmission, comprehensive surveillance studies involving collection of multiple rodent species across diverse geographic locations, seasons, and rodent species, coupled with serologic analysis, are crucial. Incorporating factors such as habitat diversity, agricultural practices, and climatic conditions into future research will also contribute to a comprehensive understanding of the ecologic niche of SFTSV.

Of note, we detected SFTSV RNA in chiggers infesting rodents that tested negative for the virus. This unexpected finding suggests a potential lack of direct rodent-to-chigger transmission. Given that chiggers feed on liquefied host skin tissue rather than blood, accidental acquisition of the virus during feeding appears less likely. Experimental studies supporting this notion demonstrated that chiggers feeding on *O. tsutsugamushi*–infected hosts failed to transmit the pathogen to their offspring (*36*). Alternatively, chiggers may acquire the virus through co-feeding with infected conspecifics and subsequently transmit the virus transovarially to their offspring, a phenomenon that has been successfully demonstrated in establishing new lines of *O. tsutsugamushi*–infected chiggers in previous research at our institute (*37*). Furthermore, reported co-positivity for SFTSV and *O. tsutsugamushi* in patients from South Korea and Myanmar (*11*,*38*,*39*) suggests a potential epidemiologic intersection between these pathogens, possibly enabled by shared vector species or overlapping habitats (*40*). The co-concurrence of these infections complicates diagnosis and underscores the importance of integrated surveillance systems to monitor these and potentially other co-circulating pathogens.

In conclusion, although the role of chiggers in SFTSV transmission remains unclear, the widespread distribution and abundance of chiggers, especially in the Asia–Pacific region, leads to frequent exposure to chiggerborne pathogens. Our findings suggest that the epidemiology of SFTSV may be more complex than previously understood, involving several potential vectors, reservoir hosts, and interactions with other pathogens. This apparent complexity underscores the need for comprehensive surveillance and research to better understand and mitigate the risks associated with this emerging infectious disease.

Appendix Additional information about comprehensive surveillance of *Bandavirus dabieense* in patients with acute febrile illness, wild rodents, and trombiculid larval mites, Thailand.
