# Supplementary material for: Comprehensive Surveillance of Severe Fever with Thrombocytopenia Syndrome Virus in Patients with Acute Febrile Illness, Wild Rodents, and Trombiculid Larval Mites, Thailand
Source: Emerg Infect Dis. 2024 Nov;30(Suppl 2):S111–9. doi: 10.3201/eid3014.240163 (PMC11559579; doi:10.3201/eid3014.240163)
Supplement: Appendix — Additional information about comprehensive surveillance of Bandavirus dabieense in patients with acute febrile illness, wild rodents, and trombiculid larval mites, Thailand. [file 24-0163-Techapp-s1.pdf]

Article DOI: https://doi.org/10.3201/eid3014.240163

EID cannot ensure accessibility for supplementary materials supplied by authors. Readers who have difficulty accessing supplementary content should contact the authors for assistance.

Comprehensive Surveillance of Severe Fever with Thrombocytopenia Syndrome Virus in Patients with Acute Febrile Illness, Wild Rodents, and Trombiculid Larval Mites, Thailand

Appendix

Acute Febrile Illness Patients

Chum Phae district is a semi-rural area located in the upper northwest of Khon Kaen province. It ranks as the fourth most economically important district in Khon Kaen with a population of 78,501 as of 2023 (Ministry of Public Health of Thailand). Chum Phae Hospital, an M1 level general hospital with a capacity of 200 beds, is renowned for delivering quality medical services to residents in the northeastern areas of Thailand. Strategically located at the junction of three provinces (Khon Kaen, Loei, and Chaiyaphum), the hospital serves as a significant healthcare provider in this region.

The acute phase samples were collected from patients who presented to the hospital with acute febrile illness (AFI) symptoms including patients that had a body temperature higher than 37.5°C and a fever persisting for more than 3 days, without prior antibiotic treatment. Additionally, samples from patients who reported experiencing high fever but presented with body temperatures lower than the specified range, likely due to self-medication with antipyretics, were also included in the study. General blood examinations, including blood cell count, blood biochemical tests, and serologic tests, were performed based on clinical necessity. After completion of the general blood examinations, anonymous, residue samples were transferred into two new sterile tubes (2 tubes/ type) and then frozen at −20°C before transported to the Center of Excellence in Clinical Virology, Chulalongkorn University and the Department of Entomology,

WRAIR-AFRIMS, in Bangkok for further analysis. The samples were stored at −80°C. Patient clinical information or discharge diagnosis were partially available for data analysis. Baseline characteristics of age, gender of the patients, body temperature at admission, and length of fever before admission were collected using a standardized form in addition to general diagnostic result reported by physicians.

In this study, residue serum samples from 2,425 AFI patients who visited Chum Phae Hospital between 2015 and 2021 were randomly and equally selected from all age groups throughout the year to investigate for the presence of SFTSV-RNA and specific antibodies against the viral nucleoprotein (Np). The study was approved by the Institutional Review Board of the Faculty of Medicine, Chulalongkorn University (IRB378–59) and the WRAIR Human Subject Protection Branch (WRAIR2386).

Wild Rodent and Ectoparasite Sampling

All procedures involving animals and ectoparasite collection were approved by the Institutional Animal Care and Use Committee of WRAIR-AFRIMS (PN21–10). The rodent and ectoparasite (chigger, tick, flea, lice) surveillance study was conducted to support Thai and United States joint military training exercises across several locations in Thailand. The majority of the collection sites were located in community settings such as schools, villages, and neighboring gardens or plantations to support the humanitarian missions accompanying the military exercises. These missions often involve building construction for local communities and outreach programs. Consequently, rodents and ectoparasites were predominantly collected from areas in close proximity to human activities rather than from densely forested sites. The pre-exercise surveillance spanned from November to February within the period of 1–2 months before the training exercise, coinciding with the cold and dry season of Thailand.

Rodents were captured using wire traps and any ectoparasites infesting rodent were collected using ether fumigation followed by thorough visual inspection. Ectoparasites were preserved in 70% ethanol and separated by orders and host. After the rodents were euthanized using carbon dioxide inhalation followed by cardiac exsanguination, their internal organs were harvested and immediately preserved in RNAlater solution (Qiagen, https://www.qiagen.com/us)

to inactivate the sample and prevent nucleic acid degradation. The samples were transported to WRAIR-AFRIMS laboratory in Bangkok on dry ice and stored at −80°C until analysis.

During the collection period between 2019 and 2023, total number of 1,299 wild rodents and insectivores were collected. To determine the potential of SFTSV transmission among rodents and their ectoparasites, our selection criteria for archived samples included: (a) samples collected during 2019 to 2022, focused on all rodents with tick, chiggers, or flea infestations (451 rodents) and (b) all rodents sampled during the 2023 surveillance period (329 rodents). Additionally, tissue samples from rodents without ectoparasite infestation were included to broaden the scope for pathogen identification (239 rodents) (Appendix Table 1). Chiggers were pre-identified to the genus or species level based on traditional morphological identification before being used for total nucleic acid extraction (*1*). Nucleic acid of the SFTSV-RNA positive chiggers were further analyzed by amplifying the cytochrome oxidase subunit I (*cox1*) gene to confirm their genus or species assignment following a previously described protocol (*2*). In total, 573 chiggers from 8 genera sampled from rodent hosts from the pre-identified locations with suspected SFTSV-RNA positive rodents were included in the study.

Detection of SFTSV-RNA using RT-qPCR

Total DNA/RNA were extracted from patient sera, rodent tissues, and chiggers using the IndiMag Pathogen Kit (Indical Bioscience, https://www.indical.com) following manufacturer instruction with additional steps of proteinase K pre-treatment (*3*). Complementary DNA (cDNA) was synthesized using iScript cDNA Synthesis Kit (Bio-Rad Laboratories, https://www.bio-rad.com). Detection was performed via RT-qPCR targeting the nonstructural protein (Ns) encoding gene in the small segment (S segment). The reaction mixture contained 2 μL of cDNA template, 3 μL of qPCR mix that consisted of 1x of SsoAdvanced Universal Probes Supermix (Bio-Rad Laboratories, https://www.bio-rad.com), 0.3 μmol each forward and reverse primers (SFTSV-S-F; ACCTCTTTGACCCTGAGTTWGACA and SFTSV-R; CTGAAGGAGACAGGTGGAGATGA), 0.4 μmol probe (SFTSV-P; FAM-TGCCTTGACGATCTTA-BHQ1) and RNase-free water to adjust final volume (*4*). Amplification cycles were performed using CFX96 Touch thermocycler (Bio-Rad Laboratories, https://www.bio-rad.com) as follows: 30 s at 95°C followed by 45 cycles of 15 s at 95°C, 30 s at

60°C, and 40 s at 45°C. The cutoff Ct was 40 cycles. All SFTSV RNA-positive samples with the Ct less than 40 were subjected for sequencing of partial gene of the S segment using a barcode-tagged sequencing method (Bionics, https://www.bionicsro.co.kr).

To assess the sensitivity of the RT-qPCR assay for SFTSV detection, two standard preparations were used: (a) tenfold serial dilutions of synthetic viral RNA transcripts of the complete SFTSV S segment and (b) viral cDNA extracts from virus stock derived from serum of an SFTSV-infected patient in South Korea (provided by Dr. Lee). These standards were employed to determine the detection limits in terms of SFTSV-RNA copy numbers. The assay demonstrated a lower detection limit of ≈10 viral RNA copies/µl. Template-free negative control was included in all RT-qPCR batches to ensure no contamination. A panel of positive control samples for dengue virus I-IV, chikungunya virus, *Orientia tsutsugamushi*, *Orientia chuto*, *Rickettsia honei*, *Rickettsia felis,* or *Rickettsia typhi* were used to determine the assay specificity.

Data Analysis

Statistical analyses were conducted using GraphPad Prism v9.1.0 and Python v3.12.1. Continuous variables are represented as mean ± SD or medians and interquartile ranges (IQR), while categorical variables are shown as frequencies and percentages. Appropriate statistical tests, such as t-tests, chi-square, Fisher exact test, ANOVA, and nonparametric tests, were used based on the data. Statistical significance was set at $\alpha = 0.05$.

References

1. Lerdthusnee K, Khuntirat B, Leepitakrat W, Tanskul P, Monkanna T, Khlaimanee N, et al. Scrub typhus: vector competence of *Leptotrombidium chiangraiensis* chiggers and transmission efficacy and isolation of *Orientia tsutsugamushi*. Ann N Y Acad Sci. 2003;990:25–35. PubMed https://doi.org/10.1111/j.1749-6632.2003.tb07333.x

2. Kumlert R, Chaisiri K, Anantatat T, Stekolnikov AA, Morand S, Prasartvit A, et al. Autofluorescence microscopy for paired-matched morphological and molecular identification of individual chigger mites (Acari: Trombiculidae), the vectors of scrub typhus. PLoS One. 2018;13:e0193163. PubMed https://doi.org/10.1371/journal.pone.0193163

3. Linsuwanon P, Auysawasdi N, Wongwairot S, Leepitakrat S, Rodkhamtook W, Wanja E, et al. Assessing scrub typhus and rickettsioses transmission risks in the Chiang Rai Province of

northern Thailand. Travel Med Infect Dis. 2021;42:102086. PubMed https://doi.org/10.1016/j.tmaid.2021.102086

4. Zhang YZ, He YW, Dai YA, Xiong Y, Zheng H, Zhou DJ, et al. Hemorrhagic fever caused by a novel bunyavirus in China: pathogenesis and correlates of fatal outcome. Clin Infect Dis. 2012;54:527–33. PubMed https://doi.org/10.1093/cid/cir804

Appendix Table 1. SFTSV RNA positivity rates in rodents by type of ectoparasite infestation

	SFTSV positive per rodent tested (% positive)				
Rodent type	2019	2020	2021	2022	2023
Rodent with ectoparasites	0/121	0/58	0/161	0/111	5/129 (3.9%)
- Chigger	0/34	0/57	0/160	0/107	2/87 (2.3%)
- Tick	0/45	0/1	-	0/3	0/1
- Flea	-	-	-	0/1	3/33 (9.1%)
- Chigger and tick	0/42	-	0/1	-	0/1
- Chigger and flea	-	-	-	-	0/7
Rodent without ectoparasite	1/26 (3.8%)	0/55	0/40	0/118	5/200 (2.5%)
Total	1/147 (0.7%)	0/113	0/201	0/229	10/329 (3%)

Appendix Table 2. SFTSV RNA positivity rates in chiggers

				N positive by host species															
Family	Genus	N chigger	N positive (%)	*Rattus tanezumi*	*Rattus exulans*	*Rattus norvegicus*	*Mus cervicolor*	*Mus caroli*	*Bandicota indica*	*Bandicota savilei*	*Maxomys surifer*	*Niviventer fulvescens*	*Berylmys berdmorei*	*Berylmys bowersi*	*Chiromyscus chiropus*	*Tupaia belangeri*	*Tupaia glis*	*Menetes berdmorei*	Average RNA level (copies/ml)
Trombiculidae	*Ascoschoengastia* sp.	178	1 (0.6%)	1	-	-	-	-	-	-	-	-	-	-	-	-	-	-	1.85 × 10^4
	Leptotrombidium sp.	73	1 (1.4%)	-	-	-	-	-	-	-	-	-	-	-	-	1	-	-	1.49 × 10^4
	Walchia sp.	163	5 (3.1%)	1	-	-	-	-	1	3	-	-	-	-	-	-	-	-	2.89 × 10^4
	Blankaartia sp.	1	1 (100%)	-	-	-	-	-	1	-	-	-	-	-	-	-	-	-	1.38 × 10^4
	Gahrliepia sp.	136	-																
	Walchiella sp.	6	-																
	Schoengastiella sp.	3	-																
	Eutrombicula sp.	2	-																
	Helenicula sp.	1	-																
Unidentified	Other mites	10	-																
	10 genera	573	8 (1.4%)	2	-	-	-	-	2	3	-	-	-	-	-	1	-	-	2.39 × 10^4

Appendix Table 3. Comparative analysis of results obtained from SFTSV-RDT with the reference SFTSV RT-qPCR (qPCR positive samples at cycle threshold (Cq) 29–37)

	SFTSV-RDT		
SFTSV RT-qPCR	Positive	Negative	Total
Positive	6[a]	2[b]	8
Negative	0	11	11
Total	6	13	19

[a] An average SFTSV RNA of the true positive samples (n = 6) was 1.69x10^6 copies/ mL (range from 2.72x10^4 to 9.86x10^6 copies/mL)
[b] SFTSV RNA of the false negative samples (n = 2) was 5.41x10^4 and - 1.29x10^6 copies/mL
Concordance = No. of samples positive by both methods + No. of samples negative by both methods)/total number of samples × 100
Sensitivity = True positive/(true positive + false negative) × 100
Specificity = True negative/(true negative + false positive) × 100
Kappa analysis was performed to compare the results of SFTSV-RDT with those of SFTSV RT-qPCR.
Concordance: 89.50; Sensitivity 100.00; Specificity: 84.60; Observed agreement: 0.89; Expected agreement: 10.05; Kappa: 0.732.

Appendix Table 4. Descriptive statistics of hematological parameters in SFTSV RNA-positive patients (Appendix Figure 1)

Parameters	Normal values	Average	SD	Median	Q1	Q3	IQR	min	max
Leukocyte	4,500–10,000 Cells/µL	7,733	3,596	7,240	6,265	8,155	1,890	3,100	18,940
Platelet count	140,000–400,000 Cells/µL	203,556	73,754	198,000	156,500	254,000	97,500	90,000	420,000
Hematocrit	M: 40%–54%, F: 37%–47%	41.6	5.7	42.5	39.6	45	5.35	25	52
Hemoglobin	M:13–17, F:12–16 g/dL	13.8	2.0	14	13	15	2	8	18
BUN	8–20 mg/dL	13	4.7	12	10	15	5	8	24
MCH	27–32 pg	28.6	3.5	28	26	31.5	5.5	20	34
MCHC	32–36 g/dL	33	0.9	33	32.5	33.5	1	31	35
Neutrophil	50%–70%	61.1	14.6	62	51	73	22	33	88
Monocyte	2%–8%	8.6	2.4	8	7	10	3	4	14
Lymphocyte	20%–40%	26.4	12.8	24	17	35.5	18.5	6	49

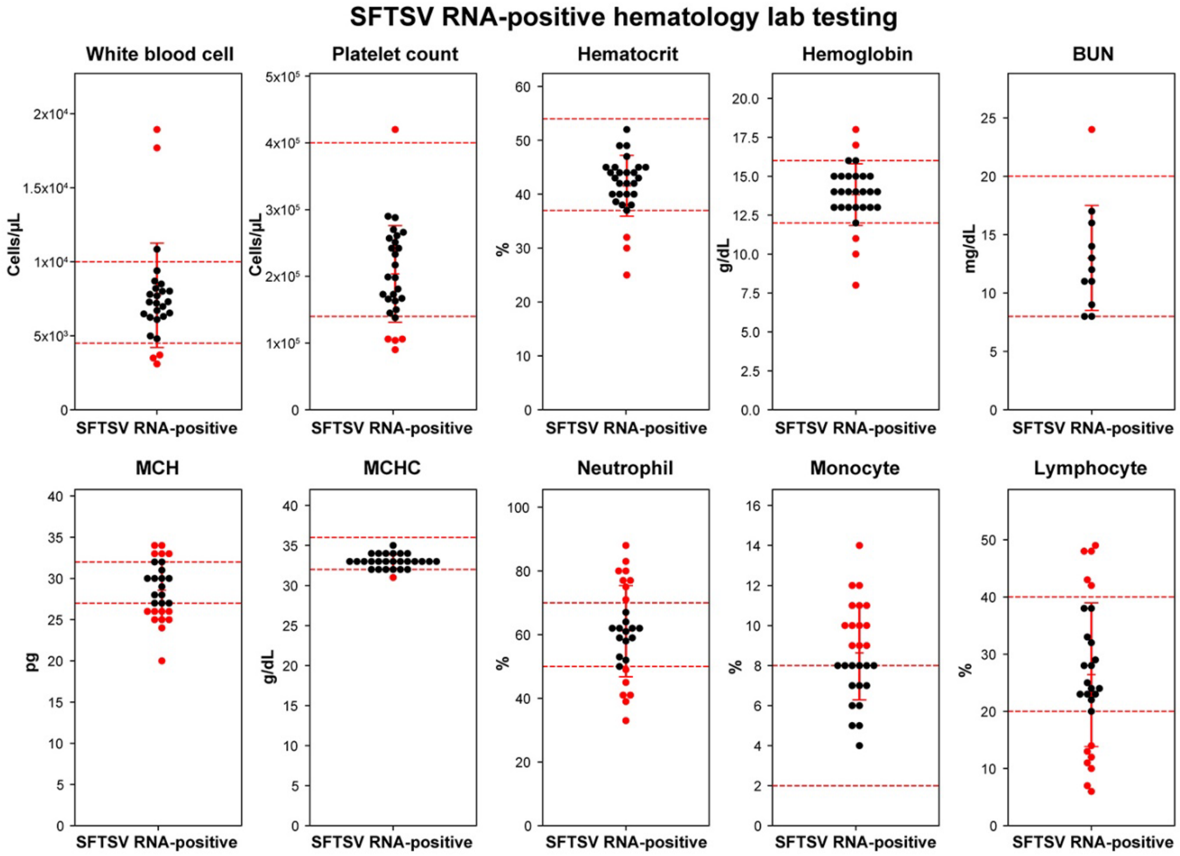

Appendix Figure 1. Hematological profiling of SFTSV RNA-positive patients. The figure shows the individual values of specific blood cell counts in SFTSV RNA-positive patients. Vertical lines indicate the normal reference range for each parameter. Filled black dots represent values within the normal range, while filled red dots represent values that fall below or above the normal range (Appendix Table 4).

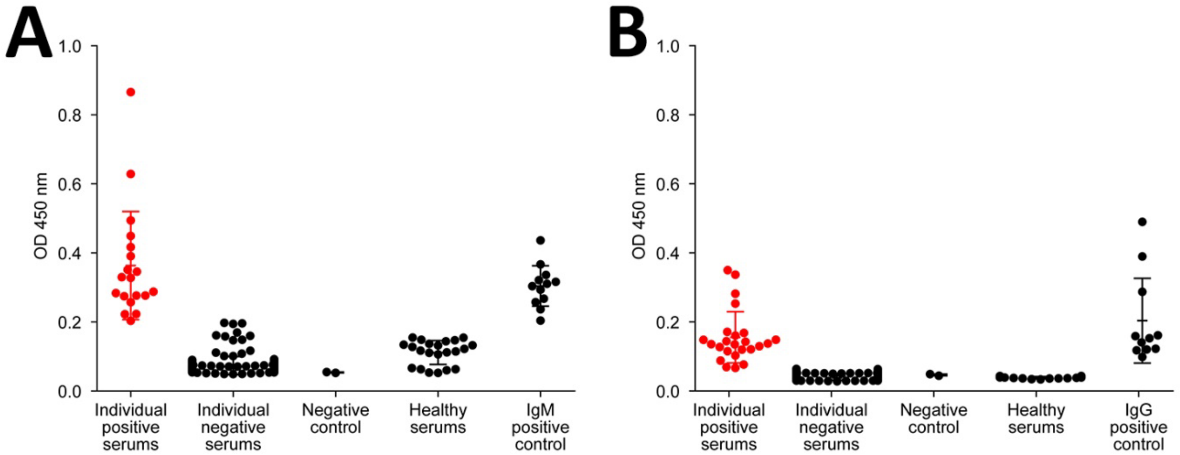

Appendix Figure 2. ELISA results showing optical density at 450 nm of the tested samples.

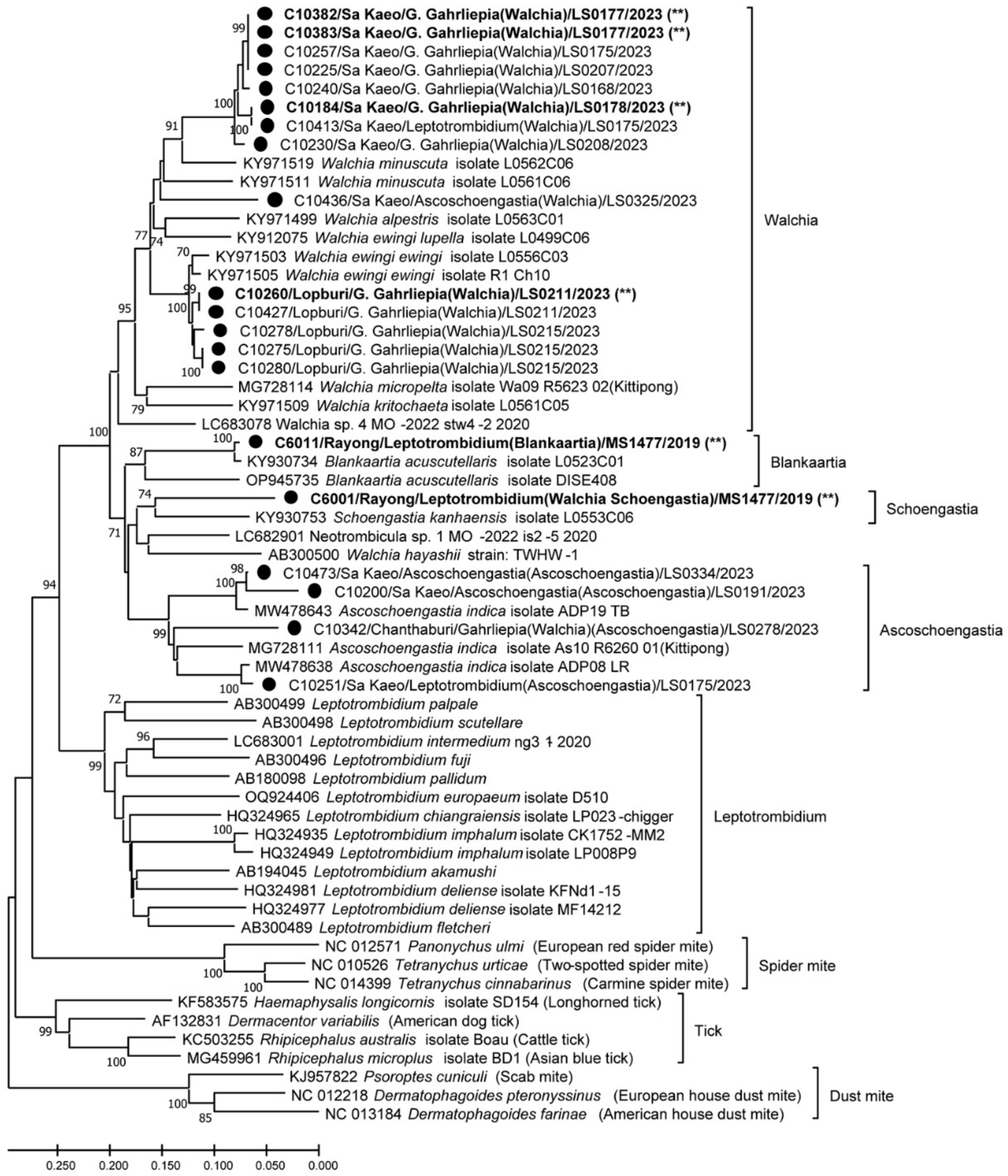

Appendix Figure 3. Phylogenetic analysis of cytochrome oxidase subunit I (*cox1*) sequences obtained from trombiculid chiggers. Sequences derived from our samples are marked with filled circles. Samples that tested positive for SFTSV-RNA are further highlighted with double asterisks. The genetic analysis of the *cox1* gene showed good correlation with the taxonomic classification at the genus level. The two chigger samples identified as different genera were collected from the same host species, indicating the possibility of co-feeding by these two genera.
